# Resemblance and clustering of mother’s and father’s psychopathology levels among Chinese parents of schoolchildren with psychiatric disorders

**DOI:** 10.1192/j.eurpsy.2020.97

**Published:** 2020-10-28

**Authors:** Yuan Gao, Yunyong Liu, Ping Wang, Xiaoxia An, Shaohe Xu, Fei Yu, Qian Chen, Yuying Li, Shuangling Wang, Jianda Lv, Guowei Pan, Ping Wang

**Affiliations:** 1 Research Center for Universal Health, School of Public Health, China Medical University, Shenyang 110122, People’s Republic of China; 2 Department of Psychiatry, Cancer Hospital of China Medical University, Liaoning Cancer Hospital & Institute, Shenyang, People’s Republic of China; 3 Institute of Chronic Disease, Shenyang Municipal Center for Disease Control and Prevention, Shenyang, People’s Republic of China; 4 Institute of Chronic Disease, Benxi Municipal Center for Disease Control and Prevention, Benxi, People’s Republic of China; 5 Institute of Chronic Disease, Anshan Municipal Center for Disease Control and Prevention, Anshan, People’s Republic of China

**Keywords:** Childhood psychopathology, family circumstances, parental psychopathology, parent–offspring associations, resemblance

## Abstract

**Background:**

Few studies have assessed the characteristics of spousal psychopathologies among parents of schoolchildren with and without psychological disorders (PD) in China.

**Methods:**

Parental symptoms were measured using the General Health Questionnaire (GHQ) in 275 mothers and 278 fathers of 298 schoolchildren with PDs diagnosed in a population survey and in 825 mothers and 834 fathers of 894 schoolchildren without PDs as a 1:3 matched comparison group. Spousal GHQ scores were compared. Childhood PD type, presence of childhood comorbidities, and multiple parental and family characteristics were examined as predictors for parental GHQ scores by multiple linear regression analyses.

**Results:**

The GHQ scores were significantly higher among mothers and fathers of children with any PD. Maternal GHQ scores were higher than paternal scores and significantly correlated with paternal GHQ scores in both groups. Spousal GHQ, personal PD history, and childhood PD comorbidity were significant independent predictors of both parents’ GHQ scores. There were also significant associations among parental chronic disease, low family income, and paternal and maternal GHQ score, as well as among low maternal education, less common disorder (LCD) prevalence in children and maternal GHQ score. The rate of GHQ score ≥3 for both parents was significantly higher in the study group than the control group (15.1 vs.7.0%).

**Conclusions:**

Parents of children with any PD type demonstrate significantly elevated psychopathologies, and psychopathology tends to occur concomitantly and resemble that of the other spouse. Screening and treatment of parental psychiatric symptoms will benefit all family members.

## Introduction

The parents of children with psychiatric disorders (PDs) are themselves at greater risk for psychiatric symptoms [1–9]. Heritability and the adverse circumstances in the family associated with childhood PDs, such as financial obligations, time commitments related to treatment, and interpersonal conflicts related to the child’s PD, are considered important causes of this elevated parental risk [1–7]. The type and severity of childhood PDs are strongly related to the stress and care burden on parents, which may trigger PD occurrence or worsening of psychopathologies among both parents, particularly parents of a child with comorbid disorders [6–10]. In turn, childhood and parental psychopathologies tend to create mutually reinforcing cycles of shared dysfunction among all family members; indeed, spouses tend to exhibit similar psychopathologies [1,2,4,11–13]. A few studies have demonstrated positive effects of the child’s treatment on maternal psychiatric symptoms, supporting the necessity for early screening and treatment of parental psychopathology [14–16].

It is estimated that about 10% of Chinese children and adolescents suffer from some form of PD [17]. However, few studies have assessed the mental health status of parents with children afflicted by PD in China, and most have focused on childhood autism [18–21] or general psychiatric disorder [22–24]. In accord with findings in other countries [1–10], most such studies have reported significantly higher parental psychiatric symptom scores compared to the norm or parents of a child without PDs [18–24]. Further, few studies have assessed the psychopathologies of parents with children affected by different types of childhood PDs, examined if the presence of comorbidities among these children worsens parental psychopathologies or evaluated whether spouses share specific features of psychopathology. Moreover, children model their attitudes, behaviors, and coping mechanisms after those of their parents, which may contribute to similarities in functioning and shared susceptibility to the same PDs. Families also share space and experiences and so are likely to encounter common stressors, which may result in similar PD outcomes for all members. However, few studies have explored the impact of mental illness in children on the mental health of parents and the effects of family demographics on this relationship. We speculated that spouses may share similar psychopathologies in part due to a shared burden as caregivers. Identification of factors influencing shared symptoms of mental illness among family members is critical for improved screening and treatment of at-risk families. This case–control study assessed the characteristics of maternal and paternal psychiatric symptoms among parents of schoolchildren with and without PDs and explored the parent–child and parent–parent mental health relationships in China.

## Materials and Methods

### Sample

The sampling procedure was described in our previous study conducted in 2008 [17], which was a cross-sectional survey of psychiatric disorders among Chinese schoolchildren aged 6–17 years living in three cities and three rural counties of Liaoning Province, China. A two-stage sampling procedure was conducted using the Strengths and Difficulties Questionnaire (SDQ) [25] as the screening instrument and the Development and Well-being Assessment (DAWBA) [26] as the diagnostic instrument. Valid SDQs were collected from 8,488 of 9,806 students, and 316 students were ultimately diagnosed with DSM-III disorders. To assess the mental health status of the parents, a self-reported version of the 12-item General Health Questionnaire (GHQ-12) [27] was completed by the mother and (or) father. For the purposes of the present study, parents and their children were excluded as candidates if neither the mother nor father returned the GHQ.

#### Study population

Population-based sample of mothers and (or) fathers whose children were diagnosed with any DSM-III-R disorder.

The parents of 316 students with DSM-III-R disorders diagnosed using the DAWBA were recruited as candidates of the “study group.” Of this parental cohort, 255 couples completed the GHQ. In addition, 20 GHQs were completed only by the mother and 23 only by the father. Finally, 275 mothers and 278 fathers of 316 students with PDs were enrolled as the study group.

#### Comparison group

Population-based sample of mothers and (or) fathers whose children were not diagnosed with any DSM-III-R disorder.

The mothers and fathers of the remaining 8,172 (8,488 − 316) students without any DSM-III-R diagnosis were defined as candidates for the comparison (control) group. Of these, 814 mothers and 820 fathers who did not return the GHQ questionnaire (including 354 couples) were excluded. The remaining mothers and (or) fathers of 7,818 students (8,172 − 354) without PDs were retained as candidates, including 6,892 couples, 460 mothers only, and 466 fathers only. Matching was done at a ratio of three mothers and (or) fathers for each mother and (or) father in the study group according to age (5-year age groups), sex (father or mother), and parental GHQ completion status (both mother and father, only mother, or only father). Finally, 765 couples, 60 mothers only, and 69 fathers only were randomly selected, for a total of 825 mothers and 834 fathers of students without PD as the control group. Data were collected between April 2010 and December 2016.

#### Development and Well-being Assessment

Details on the parent and adolescent versions of the DAWBA diagnostic interview are provided in our previous study [17]. Briefly, the Chinese version of the DAWBA was provided by the author (Prof. Robert Goodman). The following DSM-III-R disorders were diagnosed: anxiety disorders, depressive disorders, behavioral disorders, and LCDs. The anxiety disorders diagnosed included separation anxiety, specific phobia, social phobia, panic disorder, posttraumatic stress disorder, obsessive–compulsive disorder, generalized anxiety disorder, and anxiety not-otherwise-specified (NOS). The depressive disorders included major depressive disorder, other depressive disorder, and depression NOS, and the behavioral disorders included attention-deficit/hyperactivity disorder (ADHD) combined, ADHD inattentive, ADHD hyperactive, oppositional defiant disorder, conduct disorder (CD), and disruptive disorder NOS. Finally, the LCDs included eating disorders and tic disorders. Comorbidity was defined as two or more disorder types.

#### General Health Questionnaire-12

In brief, the GHQ-12 consists of 12 items, and a cut-off score of 3/12 was used to define case status (presence of symptoms) in the present study. The Chinese version of the 12-item GHQ has demonstrated satisfactory reliability, sensitivity, and validity [28].

#### Ethics statement

The study was conducted in accordance with the Declaration of Helsinki on ethical principles for medical research involving human subjects. The ethics committee of Liaoning Provincial Center for Disease Control and Prevention approved the study protocol. Parents were given an information sheet describing the study methods and goals. All parents provided written informed consent for inclusion of their children in the study.

#### Statistical analysis

Parental GHQ scores are expressed as mean ± standard deviation and case-positive rates (GHQ score ≥3) as proportions. Scores and case rates were compared between the study and control group stratified by family/parental characteristics, type of childhood PD, the presence/absence of PD comorbidity, and characteristics of parental GHQ-positive prevalence (score ≥3 by the father only, the mother only, and both parents). Receiving operating characteristic (ROC) curve analyses from studies conducted both in Taiwan [29] and mainland China [30] yielded an optimum cut-off score of 3/4 for the Chinese version of GHQ-12, supporting the choice of GHQ score ≥3 as case-positive [30]. Group and subgroup means were compared using the independent samples *t*-test or *χ* ^2^ test. Pearson correlation analysis was conducted between the GHQ scores of the mother and father in each group. We also performed stepwise multivariable linear regression analyses in which maternal or paternal GHQ score (dependent variable) was predicted by childhood PD diagnostic category (anxiety, depression, behavioral disorder, LCD, and any PD), comorbidity, age range of the child, age range of the parent, spouse’s GHQ score, education level of the spouse, family income, parental PD history, parental chronic disease (CD) history, history of divorce, and parent’s dissatisfaction with the academic performance of their child. All analyses were performed using SPSS, and all two-sided significance tests were evaluated at the 0.05 level.

## Results

The characteristics of parents and their children with PDs (the study group) are compared to the characteristics of parents and their unaffected children (the control group) in Table 1. The proportions of GHQs completed by couples, the mother only, or the father only, and the average ages of parents were comparable between study and control groups. Alternatively, divorce rate, parental PD incidence, positive CD (chronic disease) history, and parent’s dissatisfaction with child’s academic performance were significantly higher in the study group, while family income was significantly lower in the study group. Among the schoolchildren with and without PD, there were no significant differences in living area (urban vs. rural), sex ratio, presence of siblings (single child or not), and average age between study and control groups. In the study group, 57.72% of children had anxiety disorders, 27.52% depression, 28.19% behavioral disorders, and 13.09% LCDs, while 23.15% were diagnosed with two or more (comorbid) PDs. We observed significant differences in the rates of parental and maternal education level in both groups, and single child and family income level in the study group, and mothers with PD in the control group between the families in which both parents and only one parent completed the GHQ.

**Table 1.** Characteristics of study group and control group parents and children.

	Study group (children with PD, *n* = 298)		Control group (children without PD, *n* = 894)		
Characteristics	N	%	N	%	*p* value
Parents					
Only father completed GHQ	23	7.72	69	7.72	1.000
Only mother completed GHQ	20	6.71	60	6.71	
Both parents completed GHQ	255	85.57	765	85.57	
All mothers completing GHQ	275	92.28	825	92.28	
All fathers completing GHQ	278	93.29	834	93.29	
Maternal age	39.00 ± 4.95		39.03 ± 4.54		0.941
Paternal age	39.93 ± 4.97		39.60 ± 4.50		0.280
Divorced	43	14.43	52	5.82	<0.001
Maternal education level					
Primary school	55	18.46	129	14.43	0.069
Junior school	136	45.64	396	44.30	
Senior school	62	20.81	210	23.49	
College	28	9.40	127	14.21	
Missing	17	5.70	32	3.58	
Parental education level	50	16.78	122	13.65	
Primary school	139	46.64	413	46.20	0.348
Junior school	73	24.50	221	24.72	
Senior school	35	11.74	135	15.10	
College	1	0.34	3	0.34	
Missing	50	16.78	122	13.65	
Family income level					
High	88	29.53	244	27.29	<0.001
Middle	147	49.33	545	60.96	
Low	63	21.14	105	11.74	
Father with PD	15	5.03	30	3.36	0.218
Mother with PD	18	6.04	18	2.01	0.001
Father with CD	40	13.42	80	8.95	0.034
Mother with CD	36	12.08	62	6.94	0.007
Parent’s dissatisfaction with child’s academic performance	225	75.50	478	53.47	<0.001
School children					
Male student	172	57.72	518	57.94	0.946
Urban area	149	50.00	443	49.55	0.894
Single child	213	71.48	655	73.27	0.875
Average age	12.91 + 2.61		12.89 + 2.72		0.967
Psychiatric disorder					
Depression	82	27.52			
Anxiety	172	57.72			
Behavior	84	28.19			
LCD	39	13.09			
Comorbidity					
No comorbidity	229	76.85			
≥2 Diagnoses	69	23.15			
Any	298	100.00			

Abbreviations: CD, chronic disease; LCD, less common disorder; PD, psychiatric disorder.

All average maternal and paternal GHQ scores were significantly higher in the study group than the control group (*p* < 0.05), except for that of fathers of children with LCD (1.56 ± 2.38 vs. 1.08 ± 1.83, *p* = 0.138). We also found elevated GHQ scores in mothers of children with comorbid disorders compared to mothers of children with only one PD, but the difference was not found to be significant (2.72 ± 3.07 vs. 2.06 ± 2.55, *p* = 0.088), while corresponding scores were more similar between fathers of children with comorbid disorders and with only one PD (2.22 ± 2.91 vs. 1.83 ± 2.50, *p* = 0.298). Compared to fathers of children with the same type of PD, GHQ symptom scores were also significantly higher among mothers of children with LCDs (2.76 ± 2.75 vs. 1.56 ± 2.38, *p* = 0.016), and borderline significantly higher for any PD (2.20 ± 2.87 vs. 1.92 ± 2.60, *p* = 0.070) and no PD (1.24 ± 2.09 and 1.08 ± 1.83, *p* = 0.051), while scores did not differ between mothers and fathers of children with anxiety disorders, depressive disorders, behavioral disorders, or comorbid disorders.

Table 2 compares parental GHQ scores between study and control groups stratified by the demographic characteristics of both parents and children. All mean maternal and paternal GHQ scores were significantly higher in the study group than the control group (*p* < 0.001). In both groups, mean GHQ scores were significantly higher among parents with PD or CD compared to parents without PD or CD. Further, scores were significantly higher among parents dissatisfied with their child’s academic performance in both groups and highest among parents in the study group dissatisfied with their child’s academic performance. Paternal GHQ scores in both groups also increased significantly with decreasing family income, and maternal GHQ scores increased significantly with decreasing maternal education level in both groups. Parental GHQ scores increased significantly with decreasing parental education level in the study group, but not in the control group, and both maternal and parental GHQ scores were uniformly higher in the study group. Again, scores were also uniformly higher in the study group. Alternatively, scores did not differ between couples still married or divorced within the control group but did in the study group (*p* = 0.009). Again, scores were also uniformly higher in the study group. There were no within-group differences in GHQ scores between parents of rural versus urban children, male versus female children, children in different age groups, and single children versus children with siblings. Again, however, all scores were uniformly higher in the study group.

**Table 2.** Comparisons of paternal and maternal GHQ scores between and within groups stratified by parental and children’s characteristics.

	Paternal GHQ score					Maternal GHQ score				
	Control		Study			Control		Study		
	Mean	SD	Mean	SD	*p* ^1^ value	Mean	SD	Mean	SD	*p* ^1^ value
Child sex										
Male	1.04	1.78	1.91	2.04	<0.001	1.23	2.59	2.15	2.65	<0.001
Female	1.12	1.91	1.94	2.16		1.25	2.63	2.27	2.74	
*p* ^2^ value	0.157		0.906			0.920		0.347		
Urban area										
No	1.14	1.93	1.87	2.65	<0.001	1.20	2.03	2.12	2.60	<0.001
Yes	1.01	1.74	1.97	2.56		1.28	2.15	2.28	2.78	
*p* ^2^ value	0.331		0.717			0.690		0.550		
Single child										
No	1.14	2.04	2.02	2.69	<0.001	1.3	2.12	2.33	2.81	<0.001
Yes	1.07	1.78	1.86	2.56		1.22	2.08	2.15	2.64	
*p* ^2^ value	0.665		0.703			0.785		0.540		
Age										
6–10	0.87	1.71	1.65	2.95	<0.001	1.11	1.94	2.89	3.50	<0.001
11–14	1.06	1.74	2.03	2.60		1.17	1.95	2.01	2.50	
15–17	1.22	2.05	1.86	2.41		1.42	2.38	2.20	2.48	
*p* ^2^ value	0.108		0.728			0.239		0.157		
Divorced family										
Yes	1.25	1.81	2.43	3.27	<0.001	1.29	2.03	2.00	2.75	<0.001
No	1.07	1.84	1.84	2.47		1.24	2.10	2.25	2.68	
*p* ^2^ value	0.923		0.009			0.627		0.740		
Mother education level										
Primary school	1.05	1.81	2.02	2.75	<0.001	1.61	2.39	2.75	2.93	<0.001
Junior school	1.11	1.83	1.99	2.64		1.29	2.21	2.34	2.78	
Senior school	1.14	1.99	1.67	2.73		1.15	1.77	1.41	2.12	
College	0.97	1.72	1.50	1.50		0.89	1.88	1.86	2.17	
*p* ^2^ value	0.603		0.613			0.076		0.031		
Father education level										
Primary school	1.22	2.02	2.68	2.76	<0.001	1.31	2.68	2.77	2.81	<0.001
Junior school	1.10	1.83	1.82	2.57		1.38	1.82	2.36	2.88	
Senior school	1.29	2.05	1.57	2.53		1.26	1.57	1.60	2.35	
College	1.07	1.07	2.00	2.53		0.77	2.00	2.00	2.14	
*p* ^2^ value	0.002		0.177			0.038		0.115		
Family income level										
High	0.97	1.94	1.39	1.97	<0.001	1.18	2.17	2.19	2.64	<0.001
Middle	0.99	1.66	1.76	2.45		1.17	1.97	2.20	2.65	
Low	1.78	2.29	3.00	3.35		1.71	2.43	2.25	2.88	
*p* ^2^ value	<0.001		<0.001			0.085		0.721		
Father with PD										
No	1.01	1.78	1.82	2.49	<0.001	1.22	2.09	2.16	2.61	<0.001
Yes	2.79	2.32	3.67	3.77		1.79	2.15	3.17	4.09	
*p* ^2^ value	<0.001		0.006			0.166		0.203		
Mother with PD										
No	1.07	1.84	1.85	2.49	<0.001	1.18	2.02	2.03	2.55	<0.001
Yes	1.25	1.44	3.06	3.85		3.82	3.50	4.76	3.40	
*p* ^2^ value	0.603		0.057			<0.001		<0.001		
Father with CD										
No	1.03	1.79	1.65	2.35	<0.001	1.18	2.03	2.11	2.65	<0.001
Yes	1.51	2.22	3.59	3.39		1.79	2.58	2.76	2.87	
*p* ^2^ value	0.041		<0.001			0.028		0.120		
Mother with CD										
No	1.06	1.84	1.84	2.57	<0.001	1.17	2.02	2.11	2.62	<0.001
Yes	1.28	1.68	2.52	2.84		2.12	2.73	2.88	3.08	
*p* ^2^ value	0.303		0.190			0.002		0.092		
Dissatisfaction with child’s education achievement										
No	0.73	1.54	1.31	1.98	<0.001	0.78	1.66	1.70	2.19	<0.001
Yes	1.38	2.01	2.12	2.75		1.63	2.33	2.36	2.81	
*p* ^2^ value	<0.001		0.031			<0.001		0.09		

Abbreviations: CD, chronic disease; *p* ^1^ value, *p* value for *t*-test between study and control group; *p* ^2^, *p* value for χ^2^-test within group; PD, psychiatric disorder.

Multivariate analyses revealed that spousal GHQ score, personal PD history, and children’s comorbid PD were significant independent predictors of both maternal and paternal GHQ scores (Table 3), with spousal GHQ demonstrating a particularly strong effect. Lower family income (*β* = 0.091, *p* = 0.001) and chronic disease (*β* = 0.068, *p* = 0.014) were also significant independent predictors of paternal GHQ, while LCD (*β* = 0.064, *p* = 0.024) and education levels (*β* = −0.080, *p* = 0.003) were significant predictors of maternal GHQ.

**Table 3.** Multiple linear regression analyses.

	Standardized beta	SE	*p* value
Paternal GHQ			
Maternal GHQ score	0.434	15.618	0.000
Paternal PD	0.134	4.859	0.000
Family income	0.091	3.311	0.001
Child with comorbidity	0.075	2.691	0.007
Paternal CD	0.068	2.463	0.014
Maternal GHQ			
Paternal GHQ score	0.438	16.073	0.000
Maternal PD	0.194	7.144	0.000
Child with comorbidity	0.065	2.230	0.026
Maternal education l	−0.080	−2.970	0.003
Child with LCD	0.064	2.255	0.024

Abbreviations: CD, chronic disease; LCD, less common disorder; PD, psychiatric disorder.

Finally, Figure 2 shows that the rates of GHQ score ≥3 for the father only, mother only, and both parents were all significantly higher in the study group than in the control group.

## Discussion

**Figure 1.**
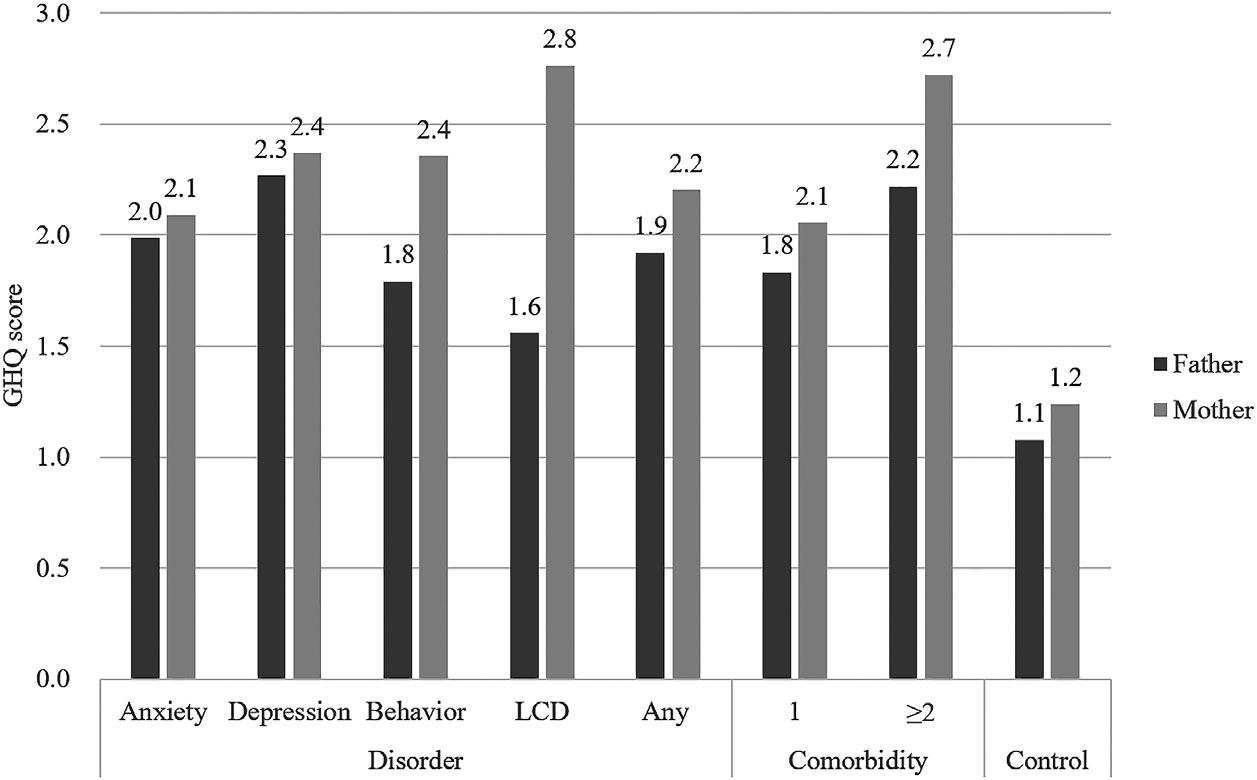
Comparison of average General Health Questionnaire scores between parents in the study and control groups stratified according to child’s psychiatric disorder type and comorbidity.

To the best of our knowledge, this is the first population-based study to examine the relationships between individual childhood psychiatric disorders (including PD comorbidity) and parental psychopathologies in China. The significantly elevated parental GHQ scores in the study group compared to a matched control group (parents of children without PDs) (Figure 1) support the premise that childhood PDs have deleterious effects on the psychological health of both mothers and fathers, in accord with previous findings [15–21] and suggesting possible “transmission” of psychopathology across generations within families [5,6]**.** Further, these psychopathologies appeared similar between couples, suggesting some mutually reinforcing driving factors within the family. As expected, GHQ scores were higher among mothers than fathers in both groups, which were consistent with previous findings that mothers played a greater role in caring the child than fathers in the family [6]. In addition, we identified multiple independent risk factors that may help in screening and treatment of these parents.

**Figure 2.**
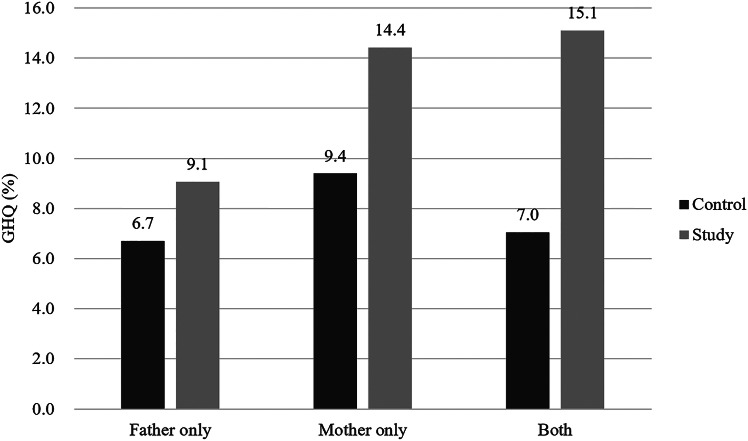
Comparison of General Health Questionnaire–positive prevalence (score ≥3) in the father only, the mother only, and both parents between study and control groups.

While the presence or absence of childhood PDs and comorbidity influenced PD symptom risk in parents, we did not find significant differences in parental GHQ scores among subgroups stratified by the specific type of childhood PD, suggesting that parental psychopathology may result from factors common to all families with such children [1–7]. The number of comorbid PDs was associated with increasing GHQ psychopathology scores among mothers and fathers in both groups. Comorbidity was also a significant predictor of maternal and paternal GHQ score according to multiple regression analyses. These data strongly suggest that parents of children with co-occurring PDs experience significantly greater stress and associated problems than parents of children with only one PD or no PD [6–10]. Many studies have shown that PD comorbidity among adolescents results in greater overall impairment, academic difficulties, mental health treatment utilization, suicidality, and conflict with parents [6,10]. The risks posed by each PD may interact, thereby increasing the probability of mental health problems among parents [31].

Maternal GHQ scores were significantly correlated with paternal scores in both the study group (*r* = 0.399, *p* < 0.001) and the control group (*r* = 0.484, *p* < 0.001), and spousal GHQ score was the strongest predictor of both maternal and paternal GHQ score. Moreover, the proportion of couples with GHQ score ≥3 (shared psychopathology) was about two times higher in the study group than the control group (15.1 vs. 7.0%). These findings indicate greater clustering and resemblance of psychopathology among parents of children with PDs compared to parents of children without PDs, in line with previous findings that the psychopathology of parents are associated with those of their children [1,2,4,11–13]. In other words, if one parent suffers from psychopathology, chances are increased that their spouse also suffers from similar psychopathology [4]. Being exposed to the same stressors may evoke psychiatric symptom development in both parents [32–34] and result in higher spousal resemblance in psychopathology [4].

Clustering and resemblance of psychopathology among parents and children present both challenges and opportunities for mental health service providers, policy makers, and family members of children with PDs. It is necessary to develop coordinated mental health systems able to provide screening, assessment, and delivery of evidenced-based treatments to afflicted children, siblings, mothers, and fathers [3]. Untreated parental psychiatric problems are likely to have a negative impact on the parents’ capacity to optimally care for and support children suffering from psychiatric illnesses; indeed, at-risk offsprings do better when parents are treated [35]. Previous studies have shown that remission of maternal depression has a positive effect on their child’s psychiatric symptoms [16], and treatment of the child’s psychopathology has a positive effect on maternal psychopathology [14], underscoring the necessity of parental screening when a child is diagnosed with a PD [4]. As expected, PD and CD histories were strong predictors of parental symptom scores, consistent with the well-established associations between psychopathology, PDs, and CDs [36]. Low family income and lower maternal education were also significant predictors of both maternal and paternal GHQ scores, which is consistent with previous reports that lower socioeconomic status is strongly associated with poor mental health status and that Chinese fathers are more sensitive to financial stress in the family [4,37,38].

This study has several limitations. First, it was not possible to determine directionality or causality between childhood PD and parental psychopathology due to the cross-sectional study design. Second, we did not gather information on the duration of adverse circumstances, such as the child’s PD and parental PD and CD. It could be that longer PD duration in children is associated with lower psychopathology due to parental adaptations or alternatively to more severe symptoms due to increasing care burden, fatigue, accrual of time and resources required, and pressures within the spousal relationship. These issues warrant future study. Third, some factors related to parental psychopathology were not assessed, such as social support, types of parent’s PDs, and life events, which may bias the results. Fourth, the school children and their parents were selected from three cities in northeast China, so it is uncertain if our findings can be generalized to other regions of China. This study also has several strengths, such as the large sample sizes of cases and controls, the inclusion of children with various psychiatric diagnoses and comorbidities, and assessment of both maternal and paternal psychopathology.

## Conclusions

This population-based study demonstrates that the parents of children with psychiatric disorders experience significantly greater psychopathology than the parents of children without PDs, particularly if the child has comorbid PDs. The severities of these symptoms are correlated between spouses and tend to occur cluster in the family. Screening and treating the parents of children with PDs will improve the treatment response and general welfare of the offspring, as well as help break the cycle of psychopathology between generations. It is necessary to conducted longitudinal studies to evaluate the factors linking childhood with adult psychopathology and test the effect of treatment of parental psychopathology on children’s PD and investigate methods for providing more effective integrated care for all family members.

## Data Availability

The datasets generated and analyzed during the current study are not publicly available because of our agreement with the participants, but are available from the corresponding author on reasonable request.
